# Supplementary figures and images for: Cost-Effectiveness Analysis of Cell Versus Egg-Based Seasonal Influenza Vaccination in Children and Adults in Argentina
Source: Vaccines (Basel). 2022 Sep 28;10(10):1627. doi: 10.3390/vaccines10101627 (PMC9612026; doi:10.3390/vaccines10101627)

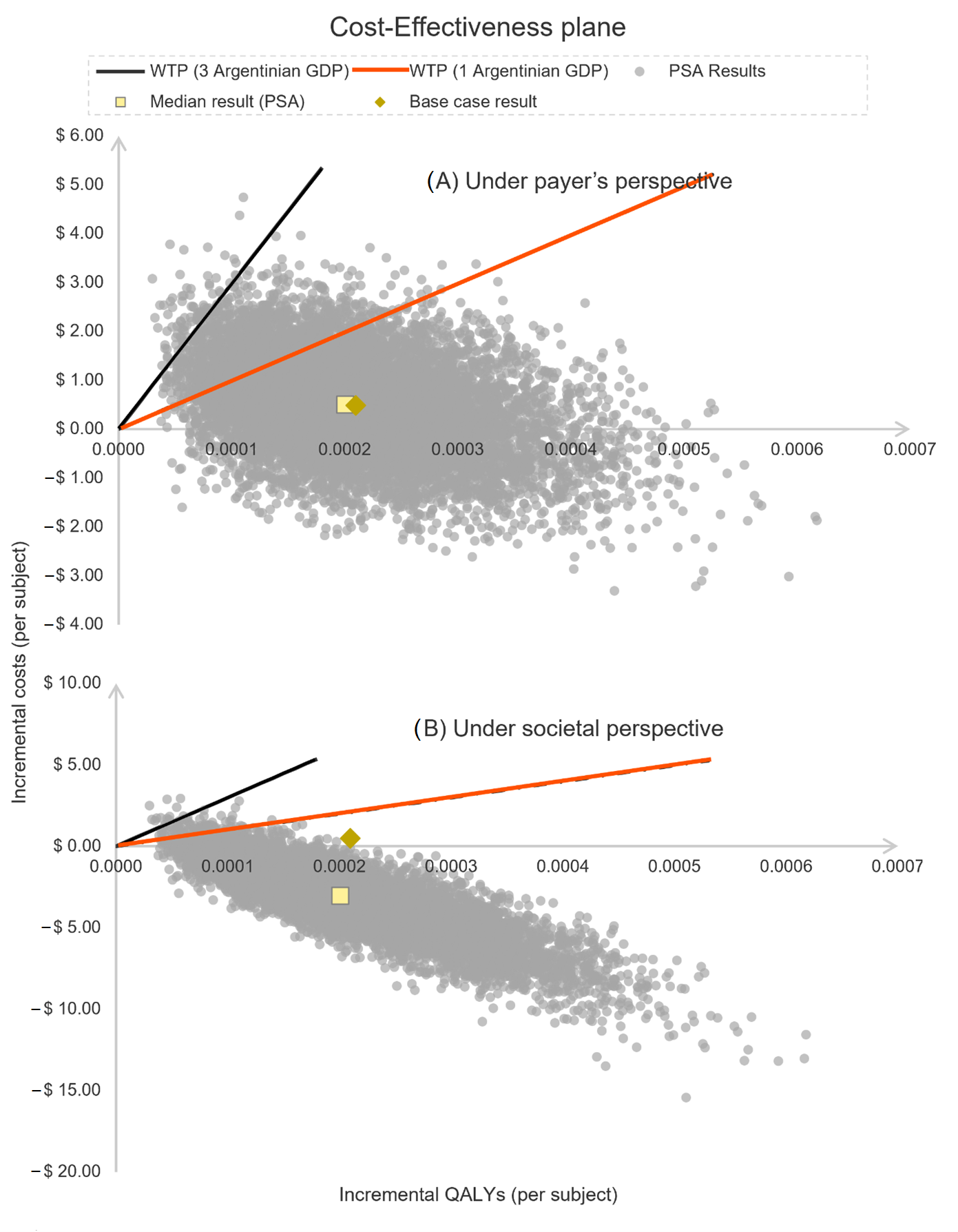

Supplement: Supplementary file 1 [file vaccines-10-01627-s001.zip › Figure S1.png]

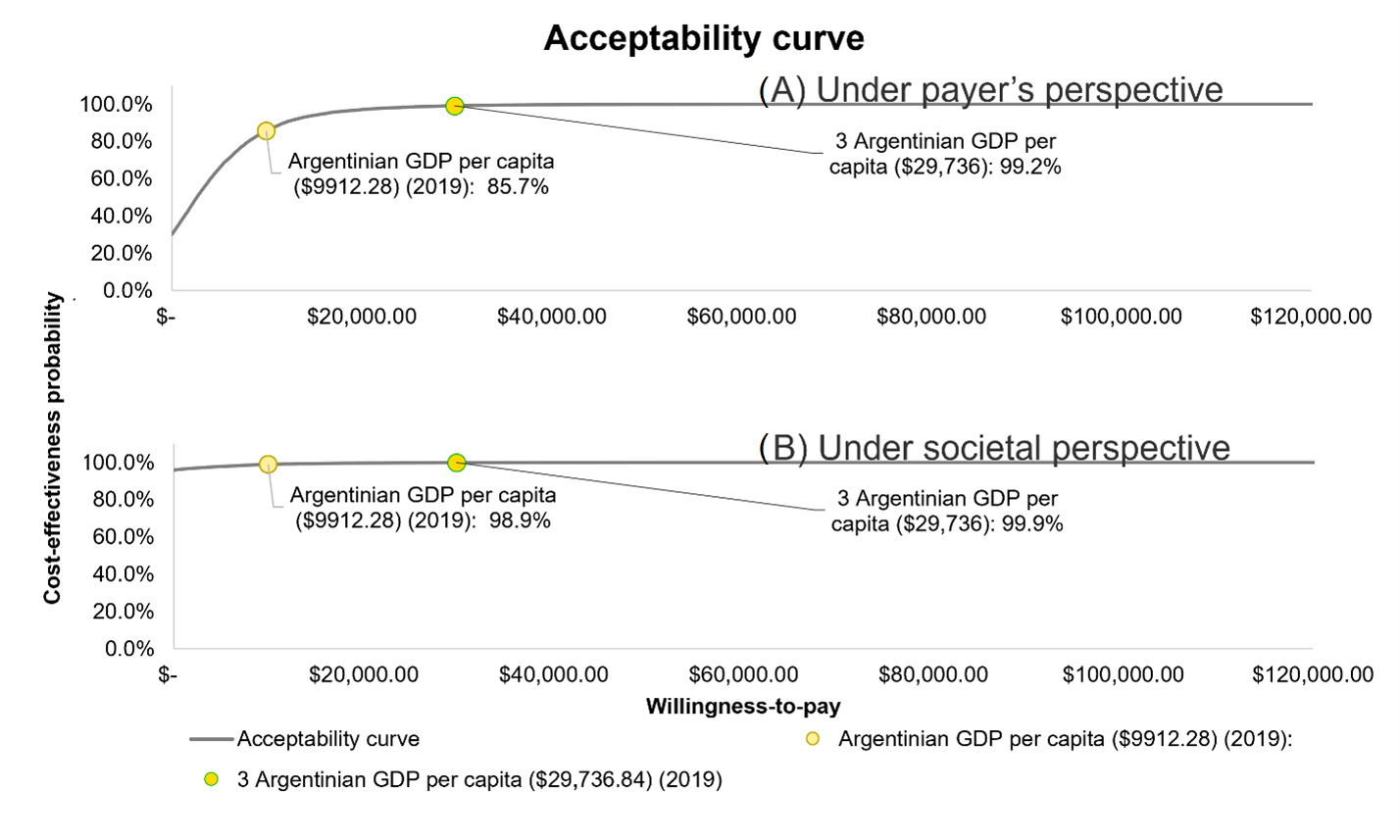

Supplement: Supplementary file 1 [file vaccines-10-01627-s001.zip › Figure S2.png]

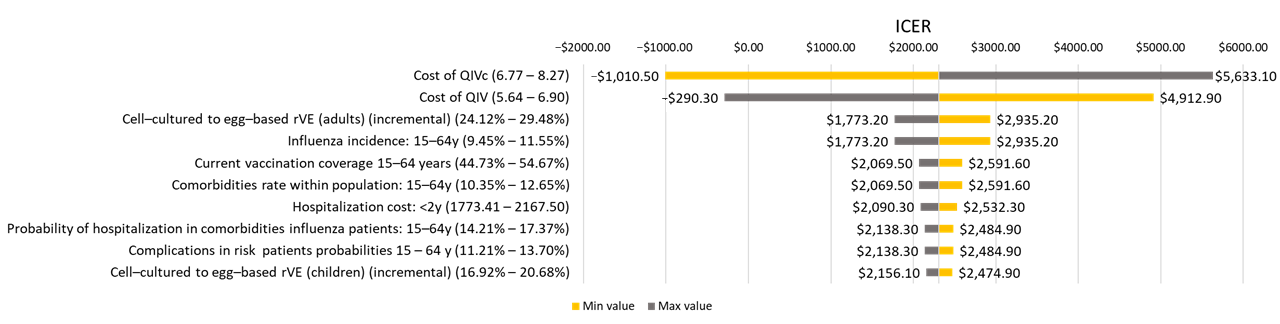

Supplement: Supplementary file 1 [file vaccines-10-01627-s001.zip › Figure S3.png]
